# CAV2 promotes the invasion and metastasis of head and neck squamous cell carcinomas by regulating S100 proteins

**DOI:** 10.1038/s41420-022-01176-1

**Published:** 2022-09-16

**Authors:** Yafei Wang, Yun Wang, Ruoyan Liu, Chunli Wang, Yi Luo, Liwei Chen, Yuchao He, Keyun Zhu, Hua Guo, Ze Zhang, Jingtao Luo

**Affiliations:** 1 Department of Maxillofacial and Otorhinolaryngology Oncology and Department of Head and Neck Oncology, Tianjin Medical University Cancer Institute and Hospital, Tianjin, 300060 China; 2 Department of Tumor Cell Biology, Tianjin Medical University Cancer Institute and Hospital, Tianjin, China; 3 Tianjin Medical University Cancer Institute and Hospital, National Clinical Research Center for Cancer, Key Laboratory of Cancer Prevention and Therapy, Tianjin’s Clinical Research Center for Cancer, Tianjin, China; 4 Department of Gynecologic Oncology, Tianjin Medical University Cancer Institute and Hospital, National Clinical Research Center for Cancer, Tianjin, China; 5 Department of Hepatobiliary Cancer, Liver Cancer Research Center, Tianjin Medical University Cancer Institute and Hospital, Tianjin, China

**Keywords:** Head and neck cancer, Metastasis

## Abstract

More than half of HNSCC patients are diagnosed with advanced disease. Locally advanced HNSCC is characterized by tumors with marked local invasion and evidence of metastasis to regional lymph nodes. CAV2 is a major coat protein of caveolins, important components of the plasma membrane. In this study, CAV2 was found to profoundly promote invasion and stimulate metastasis in vivo and in vitro. CAV2 was demonstrated to be a key regulator of S100 protein expression that upregulates the proteins levels of S100s, which promotes the invasion and migration and downregulates the expression of tumor suppressors. Mechanistically, CAV2 directly interacts with S100s in HNSCC cells, and CAV2 reduces S100A14 protein expression by promoting its ubiquitylation and subsequent degradation via the proteasome. Moreover, we discovered that CAV2 promotes the interaction between S100A14 and the E3 ubiquitin ligase TRIM29 and increases TRIM29 expression. Taken together, our findings indicate that CAV2 promotes HNSCC invasion and metastasis by regulating the expression of S100 proteins, presenting a novel potential target for anticancer therapy in HNSCC.

## Introduction

Head and neck squamous cell carcinoma (HNSCC) is a common and complex human cancer of the oral cavity, pharynx, and larynx with an annual incidence of more than 890,000 cases worldwide [1]. More than 60% of HNSCC patients are diagnosed with stage III or IV, advanced disease. Locally advanced HNSCC carries a high risk of local recurrence and distant metastasis. Although major advances have been made in improving patient outcomes, the 5-year survival rate of patients with locally advanced HNSCC remains <50% [1, 2]. Locally advanced HNSCC is characterized by tumors with marked local invasion, evidence of metastasis to regional lymph nodes, or both [1]. In 2017, the 8th edition of the Cancer Staging Manual for HNSCC contained three important changes, two of which were the addition of depth of invasion (DOI) to tumor staging in oral cavity cancers and the addition of extracapsular nodal extension (ENE) to nodal staging in nonviral HNSCC, underscoring the importance of invasion in HNSCC progression [3]. Moreover, regional neck metastasis and lymph node metastases represent ominous prognostic factors in patients with HNSCC [4, 5]. Therefore, elucidating how invasion and metastasis occur and exploiting relevant antitumorigenic therapeutic strategies are critical for improving patient outcomes.

In recent years, accumulating evidence has suggested that lipid rafts play an important role in the metastatic cascade in cancer [6]. Caveolins directly bind cholesterol and are important structural components of caveolae within the plasma membrane [7]. Caveolins are involved in multiple processes during tumor metastasis, including angiogenesis, epithelial-mesenchymal transition (EMT), migration, and the tumor microenvironment (TME) [6]. Three isoforms of the caveolin gene family are now known: caveolin-1, caveolin-2 and caveolin-3. Caveolin-1 and caveolin-2 are most abundant in endothelial cells, fibroblasts, adipocytes, pneumocytes and epithelial cells, while caveolin-3 is highly expressed in smooth and skeletal muscle [8]. Evidence for the clinical role of caveolins in cancer remains contradictory, and this evidence has mainly been derived from studies of CAV1. Few studies on the function of caveolin-2 in cancer are available, and the role of CAV2 in HNSCC remains unclear. In this study, it was found that CAV2 profoundly promotes invasion and stimulates metastasis in vivo and in vitro. Paradoxically, different classic or partial EMT phenotypes, which are regarded as hallmarks of invasion and migration, were not observed in CAV2-silenced or control HNSCC cells. A recent study found that S100 family members propagate their own expression across clonally distinct metastatic subpopulations, which could be another characteristic of migratory behavior [9].

S100 is a family of proteins approximately 10,000 Da in weight found exclusively in vertebrates. S100 proteins contain two calcium-binding sites with an EF-hand (helix–loop–helix domain)-type conformation [10]. S100 proteins play a significant role in crucial cellular behaviors in cancer, especially migration and invasion, via mechanisms such as direct interaction with cytoskeletal components [11]. Although S100 family members exhibit some sequence and structural similarity, they are not interchangeable in function [12]. Members of the S100 family exhibit distinct expression levels in each cancer type, and the role of S100s in the invasion and metastasis of HNSCC remains largely unknown. In this study, we performed many assays to evaluate how these proteins function in invasion and metastasis and found that some members drive invasive and migratory progression, while others suppress HNSCC invasion and migration. The expression of S100 family proteins is controlled by a complex regulatory network, which remains poorly understood. Interestingly, we found that the caveolin protein CAV2 is a key regulator of S100 protein expression. CAV2 was found to upregulate the proteins levels of S100s, promoting invasion and migration and downregulating the expression of members as tumor suppressors.

To delineate the mechanism by which CAV2 regulates S100 proteins, we analyzed S100 protein and mRNA levels. The results indicated that the mRNA levels were not significantly changed, while the protein levels were, which prompted us to hypothesize that CAV2 regulates S100s by stabilizing prometastatic S100 proteins and promoting the degradation of antimetastatic S100s. We verified this hypothesis by exemplifying the regulation of S100A14. In light of these findings, we reveal a novel role of caveolins in cancer through the regulation of S100 family proteins to coordinate the progression of invasion and metastasis.

## Results

### High CAV2 expression is associated with poor prognosis in HNSCC patients

**Fig. 1:**
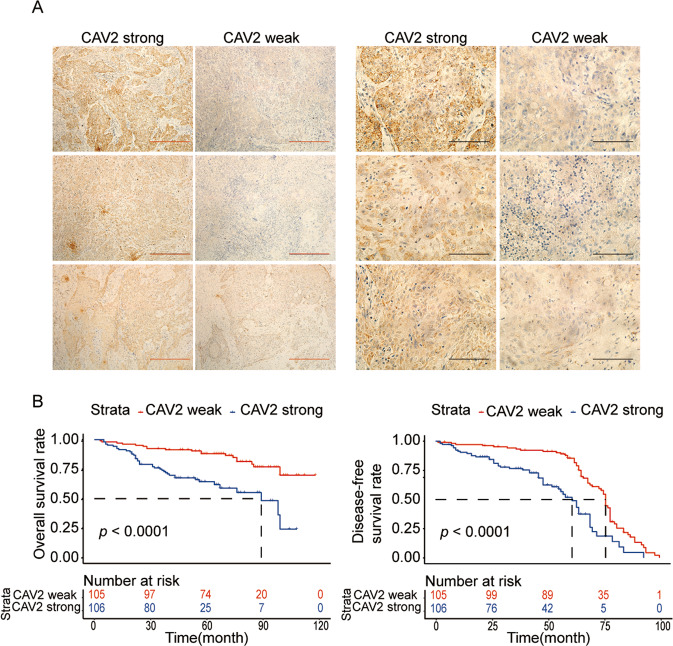
High expression of CAV2 is associated with the poor prognosis of HNSCC patients. **A** IHC analysis of CAV2 levels in 211 human HNSCC samples (scale bar, 100 μm or 400 μm). **B** Kaplan–Meier survival curve showing the correlation of overall survival and disease-free survival with the CAV2 IHC score.

To evaluate the relationship between CAV2 expression and HNSCC patient prognosis, we first analyzed the expression of CAV2 in 211 HNSCC patients by immunohistochemistry (Fig. 1A). The clinicopathological characteristics of the cohort of HNSCC patients were reported in our previous study [13]. We found by Kaplan–Meier analysis that high CAV2 expression predicted poor prognosis in HNSCC patients (Fig. 1B). Based on this, we found that the CAV2 protein is a biomarker of poor prognosis in HNSCC and speculated that CAV2 might play a role in HNSCC malignant progression.

### CAV2 promoted HNSCC migration and invasion both in vitro and in vivo

**Fig. 2:**
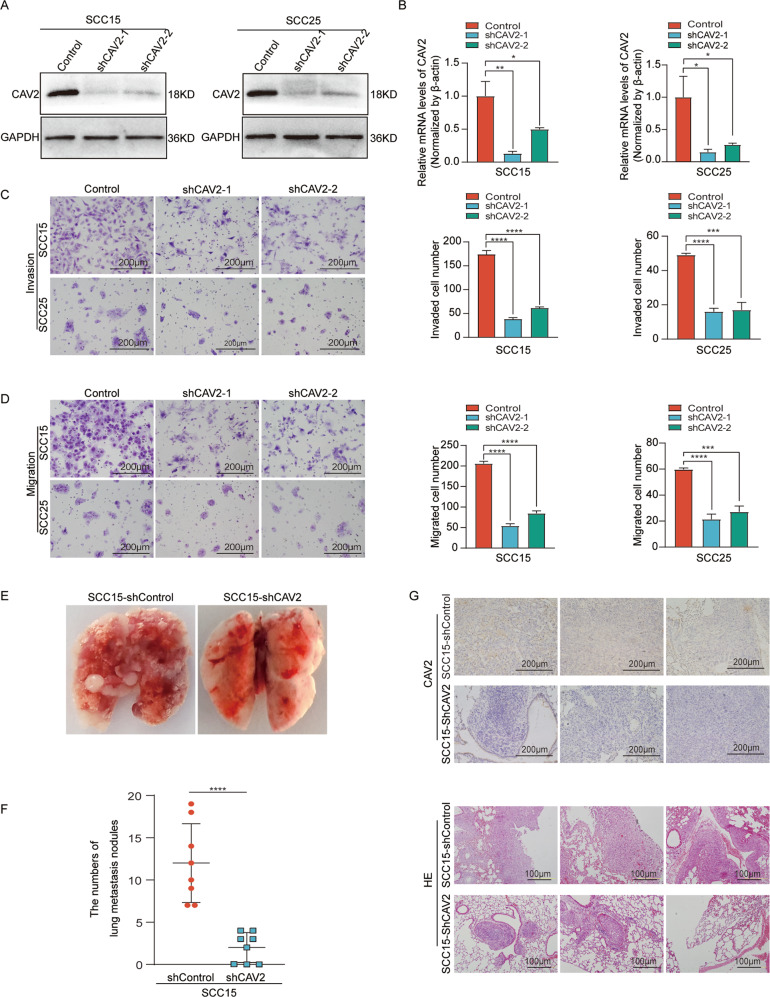
CAV2 promotes HNSCC metastasis both in vitro and in vivo. Silencing CAV2 in SCC15 and SCC25 cells by transfection with two individual CAV2 shRNAs significantly decreased CAV2 expression, as detected by western blotting (**A**) and QT-PCR (**B**). **C**, **D** The silencing of CAV2 significantly inhibited the migration and invasion of SCC15 and SCC25 cells, as evaluated using Transwell assays. (Scale bar, 200 μm). **E** Images of the macroscopic lung tissues of tail vein-injected mice. **F** The lung metastasis nodules were counted, and the data from the shCAV2 and negative control groups are summarized. ‘****’ indicates ‘*p* < 0.0001’. **G** CAV2 IHC staining and H&E staining to assess lung metastasis in tail vein-injected mice. (Scale bars, 200 μm and 100 μm).

To investigate the effect of CAV2 in HNSCC, CAV2 expression was knocked down in SCC15 and SCC25 cells by infection with virus collected from HEK293T cells transfected with a lentivirus-specific expression vector (sh-CAV2-1, sh-CAV2-2), and CAV2 expression was upregulated (CAV2-overexpression; CAV2-OE) by transfection with lentiviral plasmids. The efficiency of CAV2 downregulation and overexpression was verified by western blot analysis (Fig. 2A, Supplementary Fig. 1A) and real-time PCR (RT–PCR) (Fig. 2B, Supplementary Fig. 1B). Migration and invasion Transwell assays were performed, and the results showed that CAV2 silencing significantly inhibited the migration and invasion of HNSCC cells (Fig. 2C, D). Consistently, CAV2 overexpression significantly promoted the migration and invasion of HNSCC cells (Supplementary Fig. 1C, D). Furthermore, we evaluated the effects of CAV2 knockdown in vivo by injecting control and sh-CAV2 SCC15 cells (5 × 10^6^/mouse) via the lateral tail vein of nude mice to generate lung metastasis models. We found that CAV2 silencing significantly reduced the number of metastatic nodules compared with those of control HNSCC cells (Fig. 2E, F), and the result was verified by CAV2 immunohistochemical staining and H&E staining (Fig. 2G). The crucial role of EMT in the invasion and metastasis of cancer has been documented in many kinds of carcinoma, including HNSCC [14, 15]. However, we found that the change in CAV2 expression had no obvious effect on the protein levels of several EMT markers, including E-cadherin, N-cadherin, Vimentin, and Twist (Supplementary Fig. 1E). Together, the in vitro and in vivo results suggested that CAV2 promotes HNSCC invasion and metastasis without changing the EMT phenotype.

### The S100 protein family affects HNSCC metastasis and is regulated by CAV2

**Fig. 3:**
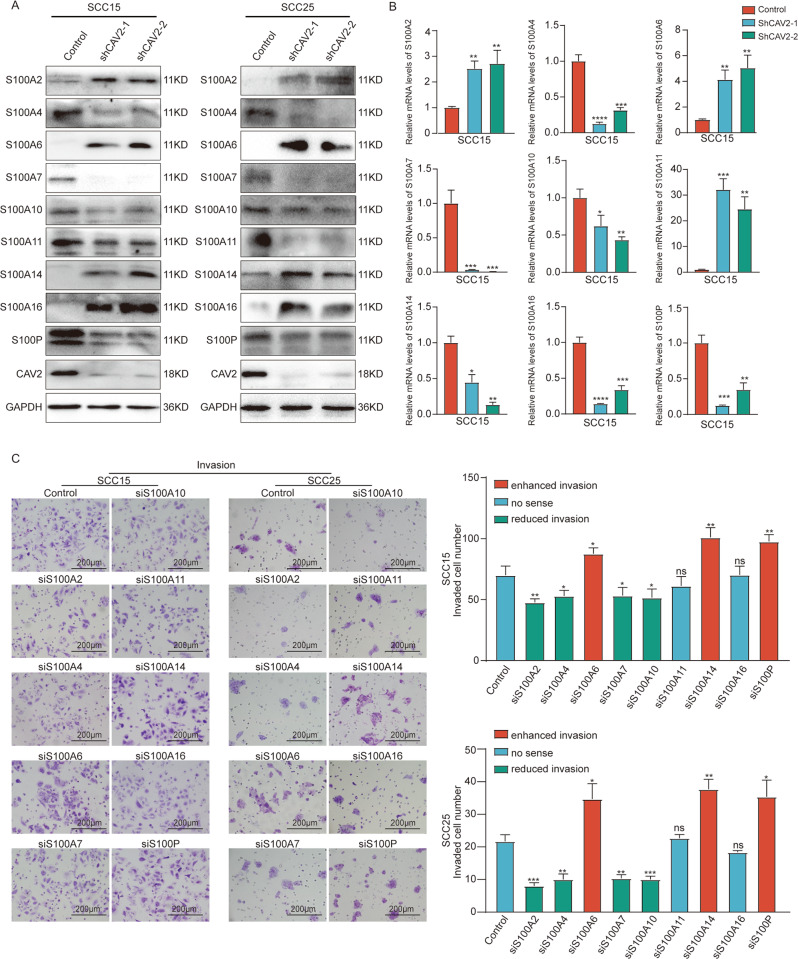
The S100 protein family affects HNSCC metastasis and is regulated by CAV2. **A** The expression of S100 family proteins in CAV2-control and CAV2-knockdown (shCAV2-1, shCAV2-2) SCC15 and SCC25 HNSCC cell lines was evaluated by immunoblotting. **B** The expression of S100 family proteins in CAV2-control and CAV2-knockdown (shCAV2-1, shCAV2-2) SCC15 cell lines was evaluated by RT–qPCR. **C** Alterations in the invasive ability of SCC15 and SCC25 cells following siRNA interference of S100 protein family members. The invasive ability of SCC15 and SCC25 cells was detected using Transwell assays. *, **, *** and **** indicate *p* < 0.05, *p* < 0.01, *p* < 0.001 and *p* < 0.0001, respectively.

To evaluate the molecular mechanisms of CAV2 in HNSCC invasion and metastasis, we performed an immunoprecipitation assay using anti-CAV2 in HNSCC cells, and a subsequent mass spectrometry assay identified multiple S100 family members in the sediment. Furthermore, S100 family proteins were found to play a significant role in crucial cellular behaviors in cancer, especially migration and invasion. Thus, we hypothesized that CAV2 promotes invasion and metastasis by regulating S100s. Then, we performed RT–qPCR and western blotting to evaluate the mRNA and protein expression levels of S100s in CAV2-deficient HNSCC cells. The protein levels of S100A2, S100A6, S100A14, and S100A16 were increased when CAV2 was silenced. In contrast, silencing CAV2 significantly decreased the protein levels of some other members of the S100 family, such as S100A4, S100A7, S100A10, S100A11, and S100P. Interestingly, the mRNA and protein levels of S100 protein family members were not consistent in either SCC15 or SCC25 cells (Fig. 3A, B, Supplementary Fig. 2A), which indicated that CAV2 most likely does not regulate S100s by transcriptional regulation. We next explored the function of S100 family members in the migration and invasion of HNSCC cells. Migration and invasion Transwell assays were performed, and the results indicated that migration and invasion were significantly inhibited when S100A2, S100A4, S100A7, or S100A10 was silenced by transfection with relevant siRNAs in SCC15 and SCC25 cells. Conversely, migratory and invasive capacities were significantly enhanced when S100A6, S100A14, or S100P was silenced in HNSCC cells. While migration and invasion did not significantly change when siS100A11 or siS100A16 was transfected (Fig. 3C, Supplementary Fig. 2B). In summary, our findings suggest that CAV2 regulates the expression of S100s and that S100 proteins function in the migration and invasion of HNSCC.

### CAV2 interacts with the S100 protein family

**Fig. 4:**
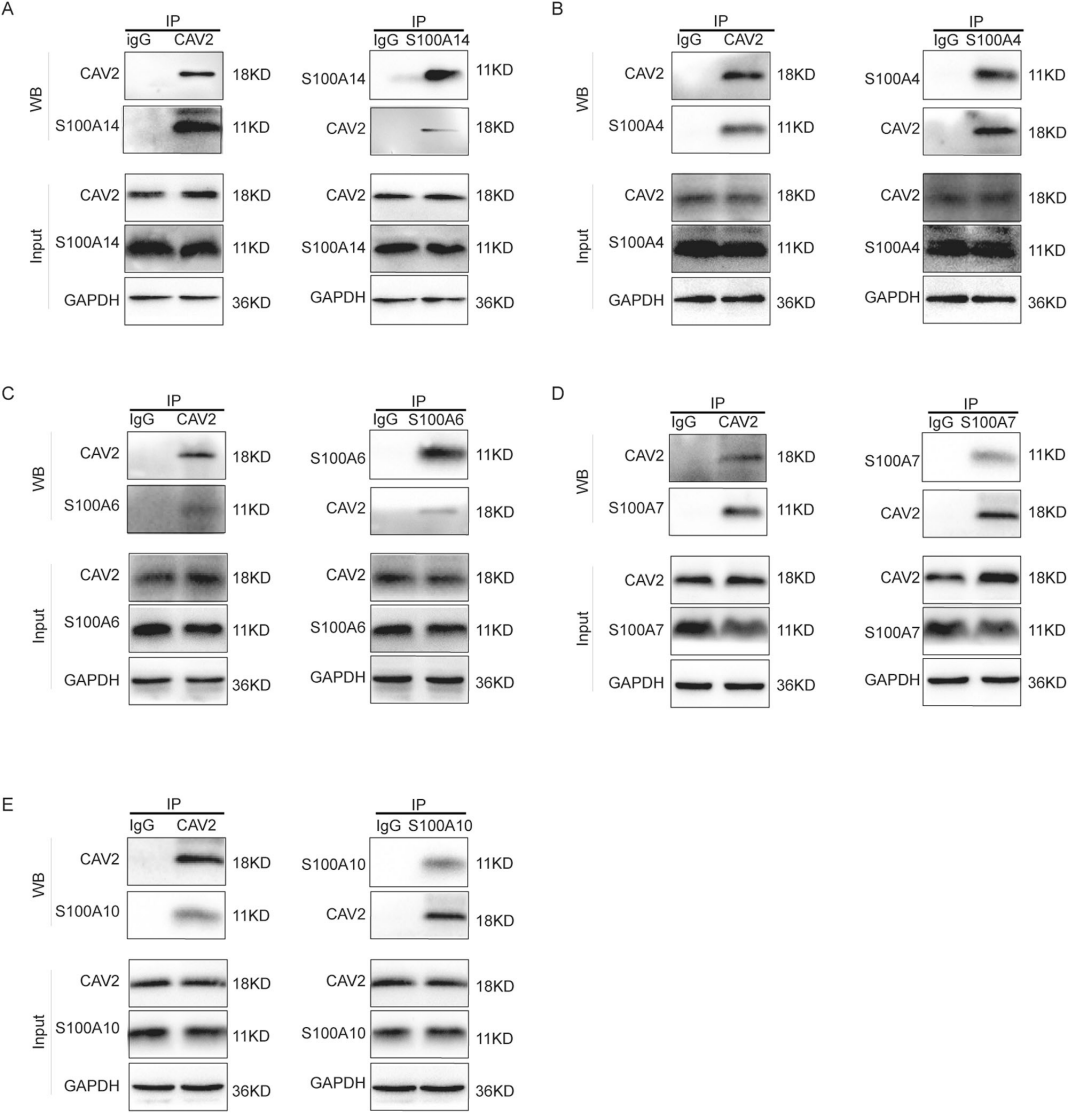
CAV2 interacts with S100 protein family members. **A**–**E** SCC15 cell lysates were subjected to immunoprecipitation (IP) with anti-S100A14, anti-S100A4, anti-S100A6, anti-S100A7, anti-S100A10 or anti-CAV2 antibodies, followed by western blotting of the immunoprecipitates with antibodies against S100A4, S100A6, S100A7, S100A10, S100A14, and CAV2, as indicated.

To confirm the results of the immunoprecipitation and mass spectrometry assays, coimmunoprecipitation experiments and reciprocal coimmunoprecipitation experiments were performed to verify the interaction between endogenous CAV2 and S100 family proteins in SCC15 cells (Fig. 4A–E). As expected, CAV2 was found to interact with S100 family members, such as S100A4, S100A6, S100A7, S100A10 and S100A14, in HNSCC cells.

### CAV2 promotes ubiquitin-proteasome-mediated degradation of S100A14

**Fig. 5:**
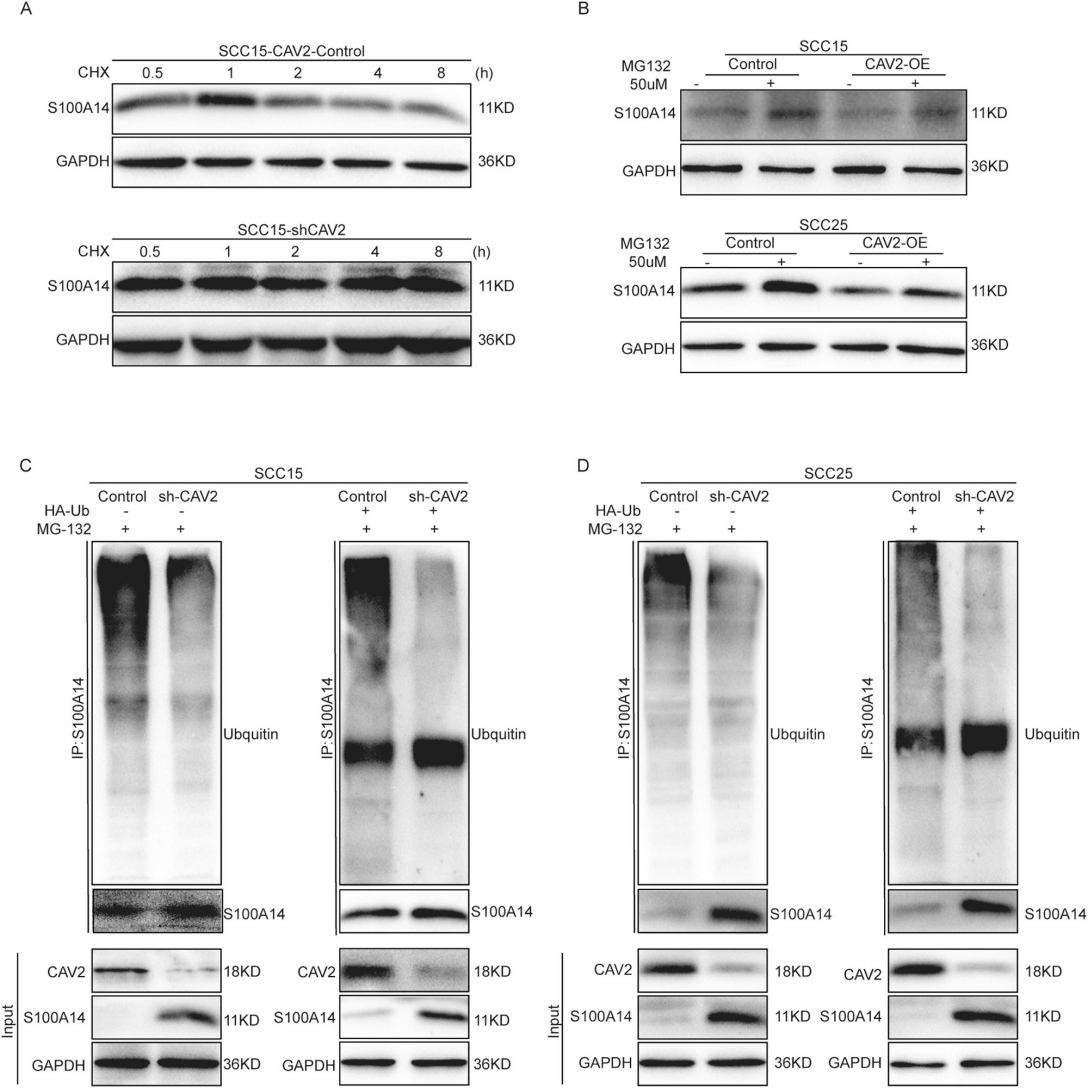
CAV2 promotes ubiquitin-proteasome-mediated degradation of S100A14. **A** S100A14 protein levels were determined by immunoblotting in vector control or shCAV2 SCC15 cells in the presence of 50 µg/mL CHX for the indicated time periods. **B** S100A14 protein levels were determined by immunoblotting in control and CAV2-overexpressing SCC15 and SCC25 cells in the presence or absence of the proteasome inhibitor MG132 (50 μM). **C**, **D** Ubiquitin-S100A14 was detected by immunoprecipitation (IP) using anti-S100A14 with a subsequent immunoblot assay with anti-ubiquitin antibody in control and shCAV2 SCC15 and SCC25 cells. HNSCC cells were transfected with or without an HA-Ub expression plasmid.

Previous studies from different teams have shown that S100A14 may play a pivotal role in the suppression of invasion and metastasis in HNSCC [16–18]. We also found that silencing S100A14 most significantly promoted HNSCC migration and invasion. We further explored the mechanism by which CAV2 regulates the S100A14 protein as an illustration to reveal how CAV2 regulates S100s. As shown by the results presented above, the S100A14 protein level increased, and the S100A14 mRNA level decreased when CAV2 was silenced in HNSCC cells. We speculate that CAV2 represses S100A14 expression by regulating its protein stability rather than inhibiting its transcription. To verify this hypothesis, we detected the effect of CAV2 silencing on S100A14 protein stability. The degradation of S100A14 was reduced in sh-CAV2 SCC15 cells treated with the protein synthesis inhibitor cycloheximide (CHX) (Fig. 5A). Then, we treated the control and CAV2-overexpressing cells with the proteasome inhibitor MG132 and found that MG132 eliminated the difference in S100A14 expression caused by exogenous CAV2 expression (Fig. 5B). Furthermore, the level of S100A14 ubiquitination was significantly reduced in sh-CAV2 HNSCC cells in ubiquitination assays (Fig. 5C, D). These results suggest that CAV2 promotes the degradation of S100A14 via the ubiquitin-proteasome pathway.

### CAV2 promotes S100A14 ubiquitination and degradation through TRIM29

**Fig. 6:**
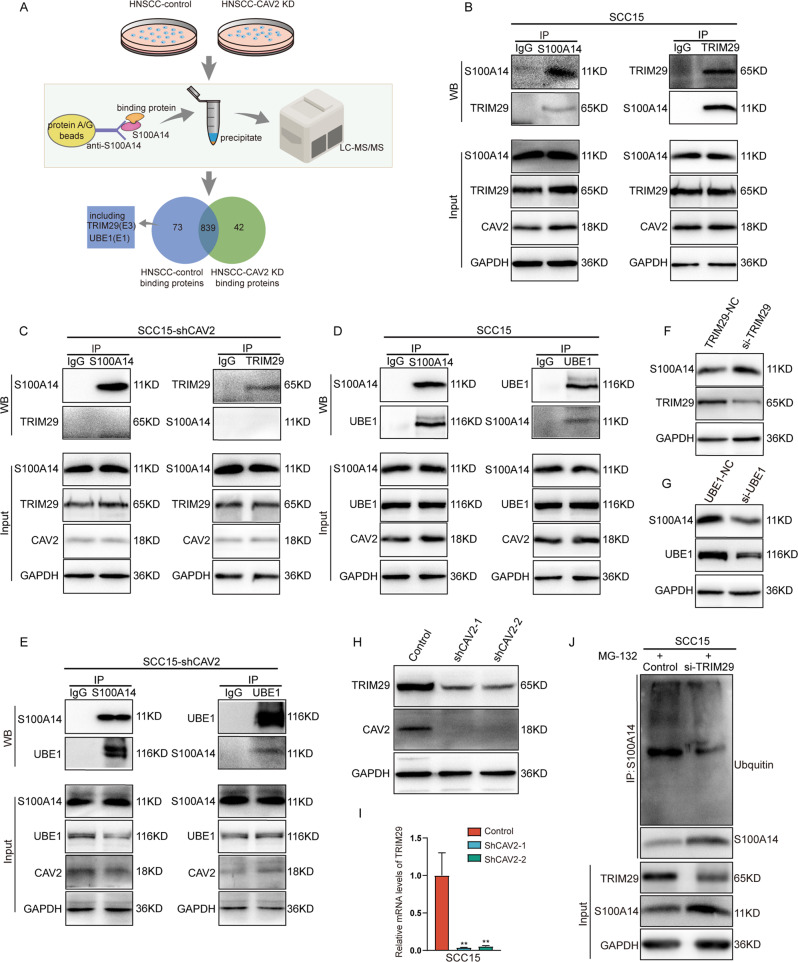
CAV2 promotes S100A14 ubiquitylation and degradation through TRIM29. **A** Immunoprecipitation assays and liquid chromatography–mass spectrometry (LC–MS) analysis of wild-type SCC15 and CAV2-deficient SCC15 cells. **B** SCC15 cell lysates were subjected to immunoprecipitation (IP) with anti-S100A14 or anti-TRIM29 antibodies, followed by western blotting of the immunoprecipitates with antibodies against S100A14 and TRIM29, as indicated. **C** shCAV2 SCC15 cell lysates were subjected to immunoprecipitation (IP) with anti-S100A14 or anti-TRIM29 antibodies, followed by western blotting of the immunoprecipitates with antibodies against S100A14 and TRIM29, as indicated. **D** SCC15 cell lysates were subjected to immunoprecipitation (IP) with anti-S100A14 or anti-UBE1 antibodies, followed by western blotting of the immunoprecipitates with antibodies against S100A14 and UBE1, as indicated. **E** shCAV2 SCC15 cell lysates were subjected to immunoprecipitation (IP) with anti-S100A14 or UBE1 antibodies, followed by western blotting of the immunoprecipitates with antibodies against S100A14 and UBE1, as indicated. **F** The protein levels of TRIM29 and S100A14 were examined by immunoblotting in SCC15 cells transfected with TRIM29-siRNA and control-siRNA for 48 h. **G** The protein levels of UBE1 and S100A14 were examined by immunoblotting in SCC15 cells transfected with UBE1-siRNA and control-siRNA for 48 h. **H**, **I** The protein or mRNA expression of TRIM29 in CAV2-control and CAV2-knockdown SCC15 cells was evaluated by immunoblotting or RT–qPCR. **J** Ubiqutin-S100A14 was detected by immunoprecipitation (IP) using anti-S100A14 with a subsequent immunoblot assay with anti-ubiquitin antibody in control and siTRIM29 SCC15 cells.

Ubiquitination is a dynamic process that involves three types of enzymes: E1 ubiquitin-activating enzymes, E2 ubiquitin-conjugating enzymes, and E3 ubiquitin ligases [19]. To further determine the enzymes involved in CAV2-mediated S100A14 degradation, we performed immunoprecipitation assays and liquid chromatography–mass spectrometry (LC–MS) analysis of wild-type SCC15 and CAV2-deficient SCC15 cells (Fig. 6A). It was found that the E1 ubiquitin-activating enzyme UBE1 and the E3 ubiquitin ligase TRIM29 interacted with S100A14, and these interactions were disrupted when CAV2 was silenced. To confirm the results of the mass spectrometry analysis, coimmunoprecipitation experiments and reciprocal coimmunoprecipitation experiments were performed to test the interaction between UBE1 or TRIM29 and S1000A14 in CAV2-deficient SCC15 and control cells (Fig. 6B–E). Consistently, TRIM29 did not interact with S100A14 when CAV2 was silenced in the coimmunoprecipitation assay. However, UBE1 still interacted with S100A14, which was inconsistent with the mass spectrometry results. Moreover, we found that the expression of S100A14 was upregulated when TRIM29 was knocked down, but UBE1 silencing did not reduce the degradation of S100A14 (Fig. 6F, G). We further evaluated the expression of TRIM29 in CAV2-deficient and control HNSCC cells and found that both the mRNA and protein levels of TRIM29 were significantly reduced when CAV2 was silenced (Fig. 6H, I). Crucially, the level of S100A14 ubiquitination was significantly reduced when TRIM29 was silenced, which supports the hypothesis that TRIM29 promotes the ubiquitylation and degradation of S100A14 as an E3 ubiquitin ligase (Fig. 6J). Collectively, these findings suggest that CAV2 promotes S100A14 ubiquitylation and degradation by promoting the interaction between TRIM29 and S100A14 and increasing TRIM29 expression.

## Discussion

Locally advanced disease, which accounts for more than 60% of HNSCC cases, is often accompanied by the invasion of surrounding structures or metastatic lymph nodes [1]. The prognosis of HNSCC is largely determined by these aggressive phenomena. To improve hazard discrimination and outcome prediction, the 8^th^ AJCC included DOI in T categorization and extranodal extension in N categorization, further suggesting the pivotal roles of invasion and metastasis in HNSCC [3]. In this study, we found that CAV2 significantly promoted HNSCC invasion and metastasis in vitro and in vivo. The results suggested that CAV2 could act as a novel target for antimetastatic therapies for HNSCC. In addition, CAV2 protein levels were inversely associated with survival, indicating that the CAV2 protein could be used as a prognostic biomarker in HNSCC.

CAV2, a major component of the inner surface of small invaginations of the plasma membrane called caveolae, participates in essential cellular functions [20]. Earlier research suggested that CAV2 levels are inversely correlated with breast cancer tumor size and that CAV2 may function as a tumor suppressor [21]. Subsequently, although CAV2 expression was observed in only 5.9% of breast cancers, the expression of this protein was found to have a prognostic impact on survival [22]. Grzegorz Sowa Host’s team observed that CAV2-knockout mice displayed reduced tumor growth and microvascular density, indicating that CAV2 may promote tumor growth by supporting tumor-induced angiogenesis [23]. In a study aiming to identify the functional genes and genetic variants associated with the prognosis of pancreatic ductal adenocarcinoma in 1070 patients, CAV2 was found to be upregulated and inversely associated with prognosis. Moreover, knockout of CAV2 suppressed tumor metastasis, and RNA-seq and bioinformatics analyses indicated that CAV2-related mRNAs were mainly enriched in pathways involved in metastasis [24]. However, this study did not further evaluate the mechanism by which CAV2 triggers metastasis. Generally, tumor epithelial cells depend on the activation of EMT to acquire distinct mesenchymal traits that confer to cells the ability to invade adjacent tissues and then to disseminate over a distance [15, 25]. Therefore, we speculated that CAV2 promotes invasion and metastasis via the activation of EMT. However, we found that silencing CAV2 in HNSCC cells did not influence the expression of primary EMT markers, prompting us to explore other potential mechanisms of CAV2 in HNSCC invasion and metastasis.

Evidence from recent decades has indicated that S100 proteins play an important role in many aspects of cancer progression, especially invasion and metastasis [26]. The function of S100s is cancer-specific and subtype-specific, and individual members can exert distinct or opposing effects. A recent study showed that S100A2 is essential for colorectal cancer metastasis [27], consistent with earlier findings in non-small-cell lung cancer [28]. S100A7 was also found to favor cancer cell migration and invasion and contribute to the formation of a proinflammatory and proangiogenic environment that favors tumor metastasis [29, 30]. Similarly, S100A10 was found to function in surface plasminogen activation, invasiveness, and the growth of pancreatic cancer cells [31]. However, the role of S100s in HNSCC remains largely unknown. In this study, attenuated invasion was observed when S100A2, S100A4, S100A7, or S100A10 was silenced, while S100A6, S100A14, and S100P knockdown in HNSCC cells enhanced invasiveness and mobility. Notably, the data from a Norwegian team showed that the loss of S100A14 expression is associated with an invasive phenotype in oral cancer [16]. Specifically, loss of S100A14 expression at the tumor-invading front was found to be associated with poor differentiation and worse prognosis [17]. In addition, a recent study proved that S100A14 suppresses the metastasis of nasopharyngeal carcinoma [18]. These findings are consistent with the data presented in this study; we found that silencing S100A14 significantly promoted HNSCC migration and invasion to the greatest extent. Accordingly, we further explored the mechanism by which CAV2 regulates S100A14 protein expression as an illustration to reveal how CAV2 regulates S100s.

To understand the possible mechanisms underlying the increase in S100A14 levels upon the loss of CAV2, we focused on the degradation machinery, as CAV2 directly interacts with S100s. The results of ubiquitination assays showed reduced ubiquitination of S100A14 in CAV2-silenced HNSCC cells, indicating that CAV2 enhances S100A14 ubiquitination and subsequent proteasomal degradation. To further elucidate the mechanism by which CAV2 promotes S100A14 ubiquitylation, we performed an immunoprecipitation assay and mass spectrometry analysis, which indicated that CAV2 promotes the interaction between S100A14 and the E3 ubiquitin ligase TRIM29. This was puzzling as E3 ubiquitin ligases are generally thought to bind substrate directly [32, 33]. However, we indeed observed that silencing CAV2 interrupted the interaction between S100A14 and TRIM29, which warrants further study. In addition, we found that CAV2 upregulates the expression of TRIM29 at both the mRNA and protein levels, which promotes ubiquitylation and subsequent proteasomal degradation as an E3 ubiquitin ligase.

In summary, these findings reveal a crucial role for CAV2 in promoting HNSCC invasion and metastasis. CAV2 interacts with S100 family members and promotes ubiquitin-proteasome-mediated degradation of S100A14 by upregulating TRIM29 and mediating the interaction between S100A14 and TRIM29. These results suggest that CAV2 is an attractive preventive and therapeutic target in HNSCC.

## Materials and methods

### Patients and tissue specimens

Human HNSCC tissue samples from patients treated at the Department of Head and Neck Surgical Oncology, Tianjin Medical University Cancer Institute & Hospital, Tianjin, China from 2009–2012 were used for the immunohistochemical evaluation of CAV2 expression and survival. The cancer stage in these patients was classified according to the guidelines of the 8th edition of the AJCC. The study was approved by the Ethics Committee of Tianjin Cancer Hospital, and informed consent was obtained before the study.

### Cell culture

Human HNSCC cells (SCC15 and SCC25) were cultured in Dulbecco’s modified Eagle’s medium (DMEM)/F12 medium (Corning, USA) supplemented with 10% fetal bovine serum (FBS; PAN-Seratech) and a 1% penicillin-streptomycin solution (PS; HyClone) in a 5% CO_2_ atmosphere at 37 °C.

### Antibodies and reagents

The sources of the antibodies and reagents used in this study were as follows: anti-CAV2 (Novus, NBP1-31116, USA), anti-GAPDH (Proteintech, 60004-1-Ig, China), anti-S100A2 (ImmunoWay, YN1118, China), anti-S100A4 (Bimake, A5363, USA), anti-S100A6 (Absin, abs137471, China), anti-S100A7 (Absin, abs139303, China), anti-S100A10 (Bimake, A5891, USA), anti-S100A11 (Affinity, DF7368, China), anti-S100A14 (Proteintech,10489-1-AP, China), anti-S100A16 (Affinity, DF4353, China), anti-S100P (Absin, abs137769, China), anti-E-Cadherin (Proteintech, 20874-1-AP, China), anti-N-Cadherin (Cell Signaling Technology, #13116, USA), anti-Twist (Cell Signaling Technology, #69366, USA), normal rabbit IgG (Cell Signaling Technology, #2729, USA), mouse IgG (Proteintech, B900620, China), and anti-Vimentin (Proteintech, 10366-1-AP, China). The secondary antibodies were HRP-conjugated Affinipure goat anti-rabbit IgG (Proteintech, SA00001-2, China) and HRP-conjugated Affinipure goat anti-mouse IgG (Proteintech, SA00001-1, China). MG132 (MedChemExpress, HY-13259, USA) was purchased from MedChemExpress. CHX (Beyotime, SC0353, China) was purchased from Beyotime.

### Western blotting

Cells were washed in cold phosphate-buffered saline (pH 6.8) three times and lysed on ice for 30 min using SDS lysis buffer supplemented with 1 mM NaF, 1 mM Na3VO4, and a 1× protease/phosphatase inhibitor cocktail. Protein denaturation was carried out at 95 °C for 10 min, followed by centrifugation at 12,000 rpm and 4 °C for at least 10 min. Equal amounts of protein (60-120 μg) were loaded onto gels, separated by SDS-PAGE, and transferred onto polyvinylidene fluoride membranes (Immobilon-P; Millipore, Billerica, MA, USA), which were blocked with 5% skim milk. Then, the membranes were incubated with primary antibodies and secondary antibodies (1:10,000). Detailed descriptions of the antibodies are provided in the Antibodies and reagents subsection.

### Plasmids, shRNAs, siRNA, and transfection

The CAV2-targeted shRNA sequences used in this study (#1, ACTGAGCCAGGATTGAATA; #2, ACCACTGTTCTGTTCATTT) and the control shRNA were purchased from GeneChem (China). The shRNAs were packaged into lentivirus by cotransfection with the packaging plasmids psPAX2 and VSVG in HEK293T cells. Lentiviruses were collected 48~72 h after transfection and frozen at −80 °C. Transfection was conducted using Lipofilter 3.0 (Hanbio, China). The siRNA and NC siRNA were purchased from Ruibo Biotechnology Co., Ltd. (China).

### RNA extraction, cDNA synthesis, and quantitative real-time PCR

Total RNA was extracted from adherent cells using TRIzol reagent (Ambion, USA). cDNA was generated using a quantitative RT–PCR kit (Takara, Japan) by reverse transcription of the RNA. Quantitative real-time PCR was carried out using predesigned primers with a QIAGEN QIAquant system according to the manufacturer’s instructions (Vazyme Biotech, China). The primer sequences used for RT–PCR analyses are shown in Table S1.

### Coimmunoprecipitation assays

Wild-type SCC15, CAV2-control, or shCAV2 SCC15 cells were plated into 10-cm plates and transfected with the indicated plasmids. Thirty-six hours after transfection, a total protein coimmunoprecipitation assay was performed with an IP/Co-IP Kit (Proteintech, China) according to the instructions.

### In vivo ubiquitination assay

The HA-Ub plasmid was transiently transfected into control and sh-CAV2 SCC15 and SCC25 cells for 36 h. Then, all cells were incubated with MG132 for 8 h and harvested in denaturing lysis buffer. Proteins in the supernatant were immunoprecipitated with an anti-S100A14 antibody to harvest ubiquitinated S100A14 or normal IgG. An immunoblotting assay was conducted to detect the S100A14 polyubiquitination level with an anti-ubiquitin antibody.

### Cell migration and invasion assays

For cell migration and invasion assays, Transwell cell culture chambers (8-μm pore size) and 24-well plates were used. The upper membranes were plated with Matrigel (BD Biosciences) as a barrier (50 μg/well) for the invasion assay. Two hundred microliters of the cell suspension at the appropriate concentration was placed into the upper chamber. After incubation at 37 °C with 5% CO_2_, the cells that passed through the chamber were fixed in 4% paraformaldehyde for 30 min and then stained. Three randomly selected views under an upright microscope were photographed, and the cells were counted for statistical analysis.

### Mass spectrometry

The control and shCAV2 SCC15 cell lysates were immunoprecipitated with anti-CAV2 primary antibodies. The wild-type SCC15, SCC25, and FaDu cell lysates were immunoprecipitated with anti-CAV2 primary antibodies. The wild-type SCC15 and shCAV2 SCC15 cell lysates were immunoprecipitated with anti-S100A14 primary antibodies. TMT proteomics analysis and LCMS with the following procedures were used to identify proteins from the samples and performed by Qinglian Biotech Co., Ltd. (Beijing, China).

### Animal studies

BALB/c female nude mice (4–5 weeks old) were purchased and maintained under specific-pathogen-free conditions. All animal experiments adhere to the National Institutes of Health Guide for the Care and Use of Laboratory Animals. For the experimental lung metastasis model, 1.0 × 10^6^ control or shCAV2 SCC15 cells were suspended in 100 µl of PBS for injection through the lateral tail vein of the mice. Each group contained more than 8 mice, and the mice were sacrificed 8 weeks after injection. Then, paraformaldehyde fixation of the mouse lung tissues was performed, and the nodules were counted. The mouse lung tissues were embedded in paraffin and subjected to hematoxylin and eosin (H&E) staining and immunohistochemistry analysis.

### Immunohistochemistry analysis

The tissue samples were paraffin-embedded and cut into 4-µm-thick sections. These pathological sections were heated overnight, deparaffinated in xylene, and then rehydrated in graded ethanol solutions. The sections were boiled for 3 min in sodium citrate buffer for antigen retrieval. Then, 3% H_2_O_2_ was used to eliminate the internal peroxidase activity. Each section was incubated with the primary antibody at 4 °C overnight, followed by staining with the HRP-conjugated secondary antibody. The anti-CAV2 antibody was used for incubation at a dilution of 1:100. Scoring was performed according to the percentage and intensity of positively stained cells. A value of 0 indicated negative staining, 1 indicated weakly positive staining, 2 indicated moderately positive staining, and 3 indicated strongly positive staining. Estimates were made based on the percentage of positively stained tumor cells ranging from 0 to 100. The intensity was multiplied, and the proportion score was the final protein expression score. Two independent pathologists viewed and scored the stained sections in a blinded manner based on both the intensity and the percentage of positively stained cells. The patients were grouped by the median final score.

### Statistical analyses

Differences between two groups were analyzed using Student’s *t*-test. The correlation between factors was assessed by the chi-square test and linear regression analysis. The univariate Kaplan–Meier method and multivariate Cox proportional hazards model were used to analyze the prognostic results. R version 3.5.1 was used to evaluate the data.

## Supplementary information

Supplementary Figure legends

Supplementary Table 1

Supplementary Table 2

Supplementary Figure 1

Supplementary Figure 2

Original Data File

## Data Availability

The datasets analysed during the current study are available in the manuscript and supplementary materials.
