# Supplementary material for: CAV2 promotes the invasion and metastasis of head and neck squamous cell carcinomas by regulating S100 proteins
Source: Cell Death Discov. 2022 Sep 16;8:386. doi: 10.1038/s41420-022-01176-1 (PMC9481523; doi:10.1038/s41420-022-01176-1)
Supplement: Supplementary file 1 — Supplementary Figure legends [file 41420_2022_1176_MOESM1_ESM.docx]

**Supplementary figure legends**

Supplementary Figure 1 (A-B) The construction of CAV2-control and CAV2-overexpressing (CAV2-OE) SCC15 and SCC25 HNSCC cell lines. Data were expressed as mean ± SD for triplicate experiments. * indicates p < 0.05, ** indicates p < 0.01; Student’s t test. (C-D) The overexpression of CAV2 promoted the migration and invasion of SCC15 and SCC25 cells, as evaluated using Transwell assays. (Scale bar, 200 μm). (E) The protein levels of several EMT markers, including E-cadherin, N-cadherin, Vimentin and Twist, were examined by immunoblotting in the control and sh-CAV2 SCC15 and SCC25 cell lines. Data were expressed as mean ± SD for triplicate experiments. ** indicates p < 0.01, *** indicates p < 0.001; Student’s t test.

Supplementary Figure 2 (A) The expression of S100 protein family members in CAV2-control and CAV2-knockdown (shCAV2-1, shCAV2-2) SCC25 cell lines was evaluated by RT–qPCR. Data were expressed as mean ± SD for triplicate experiments. ** indicates p < 0.01, *** indicates p < 0.001; Student’s t test. (B) Alterations in the migratory ability of SCC15 and SCC25 cells following siRNA interference of S100 protein family members. The migratory ability of SCC15 and SCC25 cells was detected using Transwell assays. Data were expressed as mean ± SD for triplicate experiments. *, **, *** and **** indicate p < 0.05, p < 0.01, p < 0.001 and p < 0.0001, respectively; ns indicates no significance. Student’s t test.
