# Supplementary material for: CAV2 promotes the invasion and metastasis of head and neck squamous cell carcinomas by regulating S100 proteins
Source: Cell Death Discov. 2022 Sep 16;8:386. doi: 10.1038/s41420-022-01176-1 (PMC9481523; doi:10.1038/s41420-022-01176-1)
Supplement: Supplementary file 2 — Supplementary Table 1 [file 41420_2022_1176_MOESM2_ESM.doc]

| **Supplementary Table 1** Primers sequences used for RT-PCR analyses | |
| --- | --- |
| CAV2-F | AAGACCTGCCTAATGGTTCTGC |
| CAV2-R | CTCGTACACAATGGAGCAATGAT |
| S100A2-F | CCAGCTTTGTGGGGGAGAAA |
| S100A2-R | TGAGTGCCAGGAAAACAGCA |
| S100A4-F | CTAAAGGAGCTGCTGACCCG |
| S100A4-R | TGTCCCTGTTGCTGTCCAAG |
| S100A6-F | GAAGGAGCTCACCATTGGCT |
| S100A6-R | CACCTCCTGGTCCTTGTTCC |
| S100A7-F | ACCTCGCCGATGTCTTTGAG |
| S100A7-R | CCATGGCTCTGCTTGTGGTA |
| S100A10-F | AAAAGACCCTCTGGCTGTGG |
| S100A10-R | AATGGTGAGGCCCGCAATTA |
| S100A11-F | GGTGTCCTTGACCGCATGAT |
| S100A11-R | AGGAAGGAGTCATGGCAAGC |
| S100A14-F | CTGACCCCTTCTGAGCTACG |
| S100A14-R | TTCTCTTCCAGGCCACAGTT |
| S100A16-F | GCTCAAGGTGCTGATGGAGA |
| S100A16-R | CAGCCACGAACACGATGAAC |
| S100P-F | AGGTGCTGATGGAGAAGGAG |
| S100P-R | ATGGCTCTGCAGGAATCTGT |
| β-actin-F | TCATCACCATTGGCAATGAG |
| β-actin-R | CACTGTGTTGGCGTACAGGT |
