# Supplementary material for: CAV2 promotes the invasion and metastasis of head and neck squamous cell carcinomas by regulating S100 proteins
Source: Cell Death Discov. 2022 Sep 16;8:386. doi: 10.1038/s41420-022-01176-1 (PMC9481523; doi:10.1038/s41420-022-01176-1)
Supplement: Supplementary file 3 — Supplementary Table 2 [file 41420_2022_1176_MOESM3_ESM.doc]

| **Supplementary Table 2** SiRNA sequences used in this study | |
| --- | --- |
| siS100A2 | CTTCCAGGAGTATGCTGTT |
| siS100A4 | TGAGCAACTTGGACAGCAA |
| siS100A6 | GGCTGATGGAAGACTTGGA |
| siS100A7 | CCAGACGUGAUGACAAGAUTT |
| siS100A10 | CCATGATGTTTACATTTCA |
| siS100A11 | GTCCTTGACCGCATGATGA |
| siS100A14 | CTCAGGAATTCAGTGATGT |
| siS100A16 | GAACCTGGATGCCAATCAT |
| siS100P | CGTCTGCCTGTCACAAGTA |
| siTRIM29 | CCAACTACTTCAGCATGGA |
| SiUBE1 | ACACGGAGCGCATCTATGA |
