# Supplementary figures and images for: CAV2 promotes the invasion and metastasis of head and neck squamous cell carcinomas by regulating S100 proteins
Source: Cell Death Discov. 2022 Sep 16;8:386. doi: 10.1038/s41420-022-01176-1 (PMC9481523; doi:10.1038/s41420-022-01176-1)

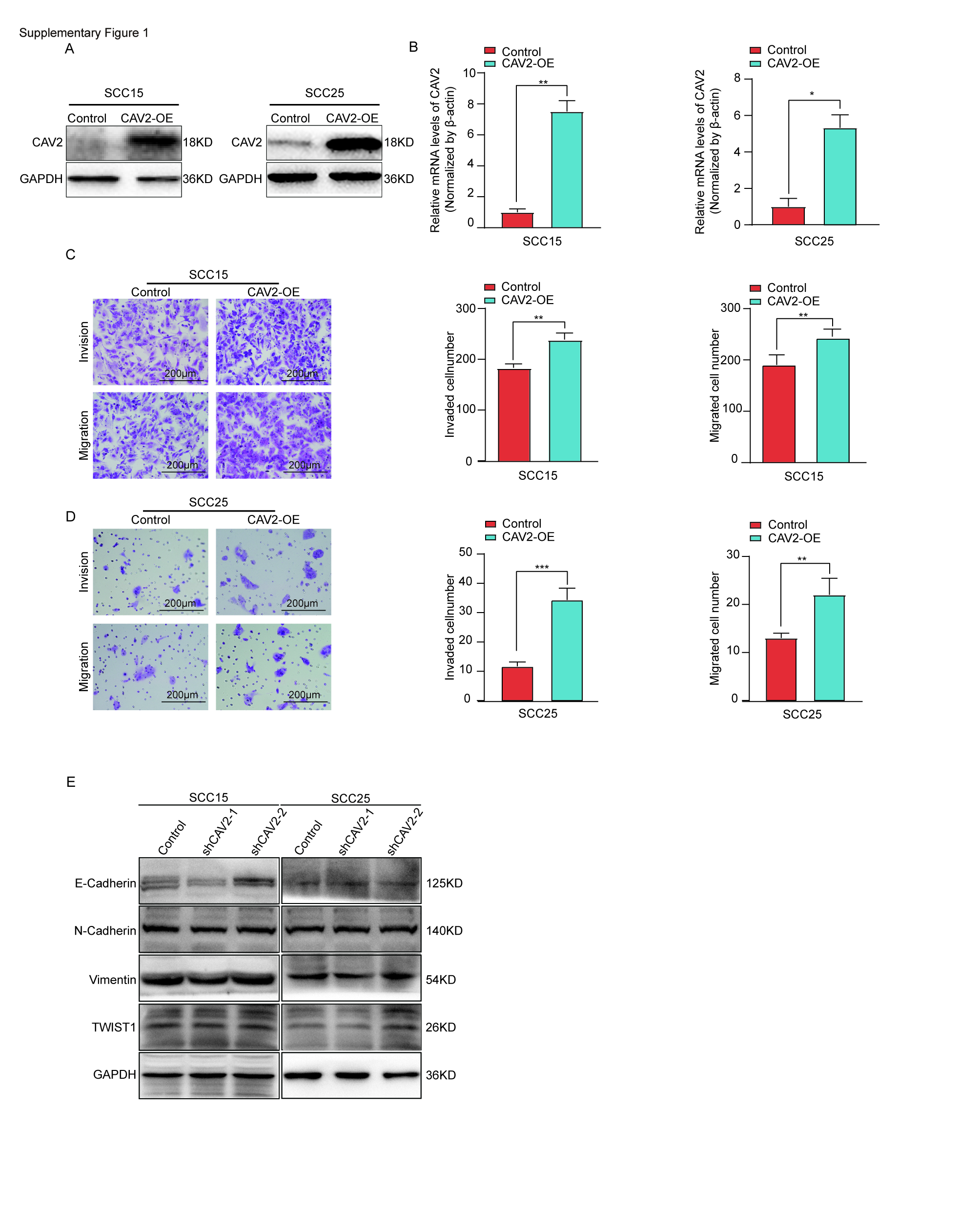

Supplement: Supplementary file 4 — Supplementary Figure 1 [file 41420_2022_1176_MOESM4_ESM.tif]

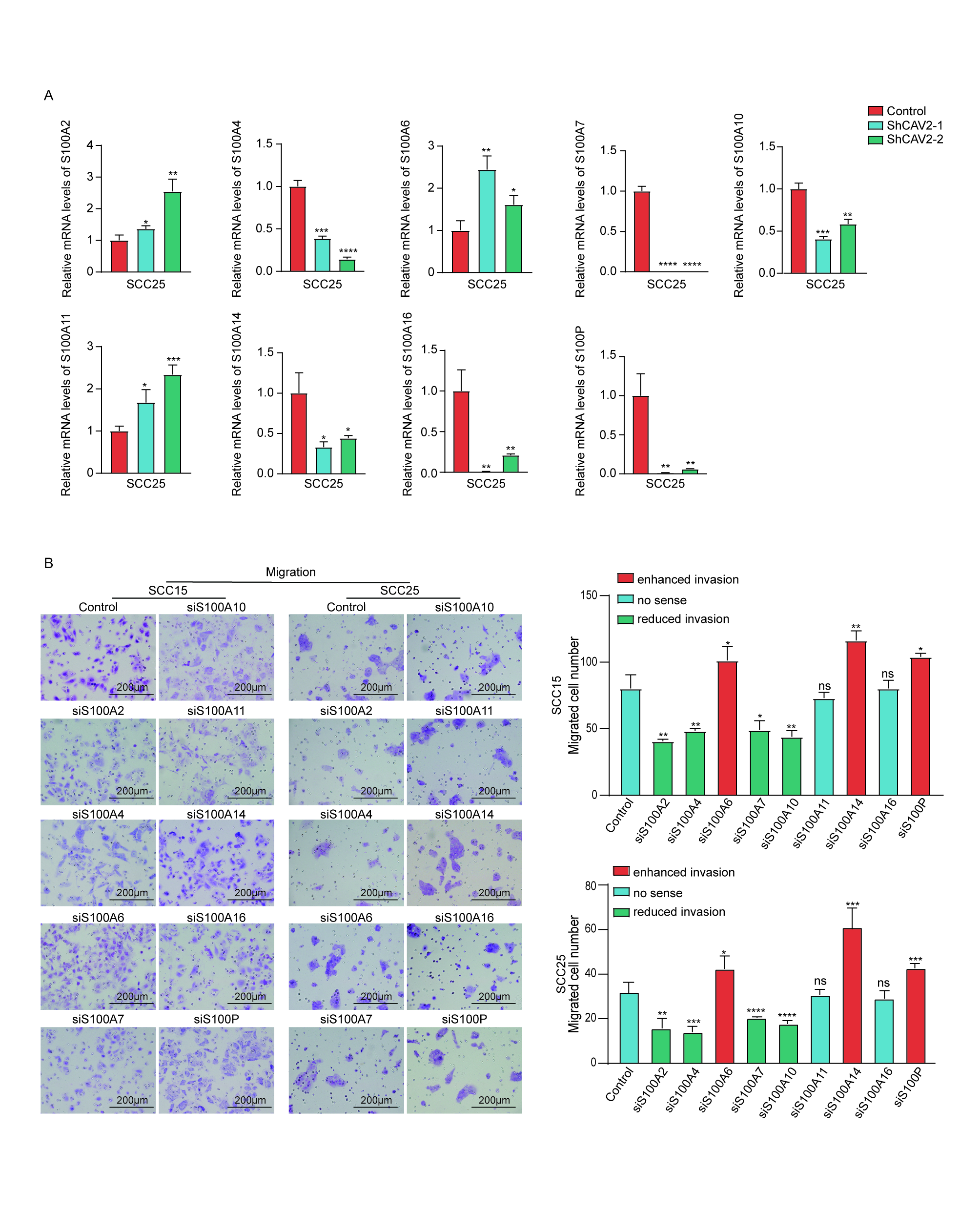

Supplement: Supplementary file 5 — Supplementary Figure 2 [file 41420_2022_1176_MOESM5_ESM.tif]
